# Characterization and correction of the false-discovery rates in resting state connectivity using functional near-infrared spectroscopy

**DOI:** 10.1117/1.JBO.22.5.055002

**Published:** 2017-05-10

**Authors:** Hendrik Santosa, Ardalan Aarabi, Susan B. Perlman, Theodore J. Huppert

**Affiliations:** a University of Pittsburgh, Department of Radiology, Pittsburgh, Pennsylvania, United States; b Universite de Picardie Jules Verne, Department of Medicine, Amiens, France; c University of Pittsburgh, Department of Psychiatry, Pittsburgh, Pennsylvania, United States; d University of Pittsburgh, Departments of Radiology and Bioengineering, Clinical Science Translational Institute, and Center for the Neural Basis of Cognition, Pittsburgh, Pennsylvania, United States

**Keywords:** functional near-infrared spectroscopy, resting state, statistical models, brain imaging

## Abstract

Functional near-infrared spectroscopy (fNIRS) is a noninvasive neuroimaging technique that uses low levels of red to near-infrared light to measure changes in cerebral blood oxygenation. Spontaneous (resting state) functional connectivity (sFC) has become a critical tool for cognitive neuroscience for understanding task-independent neural networks, revealing pertinent details differentiating healthy from disordered brain function, and discovering fluctuations in the synchronization of interacting individuals during hyperscanning paradigms. Two of the main challenges to sFC-NIRS analysis are (i) the slow temporal structure of both systemic physiology and the response of blood vessels, which introduces false spurious correlations, and (ii) motion-related artifacts that result from movement of the fNIRS sensors on the participants’ head and can introduce non-normal and heavy-tailed noise structures. In this work, we systematically examine the false-discovery rates of several time- and frequency-domain metrics of functional connectivity for characterizing sFC-NIRS. Specifically, we detail the modifications to the statistical models of these methods needed to avoid high levels of false-discovery related to these two sources of noise in fNIRS. We compare these analysis procedures using both simulated and experimental resting-state fNIRS data. Our proposed robust correlation method has better performance in terms of being more reliable to the noise outliers due to the motion artifacts.

## 1 Introduction

Spontaneous (resting state) functional connectivity (sFC) has become a critical tool for cognitive neuroscience. Interest from the neuroscientific community stems from understanding task-independent neural networks in addition to comparisons of healthy and disordered brain function.^1^^,^^2^ Recent studies have provided convincing evidence that the activity from neural regions that tend to correlate in response to a stimulus or task also spontaneously correlate even while an individual is at rest.^3^ Studies have demonstrated that spontaneous activity has a high degree of spatiotemporal organization^1^^,^^3^^–^^13^ with much of this structure being remarkably consistent across time^10^^,^^14^ and individuals.^3^ Such spontaneous activity is present in nearly all brain states (including under anesthesia and some sleep states)^15^^,^^16^ and mostly follows known anatomical connections.^17^^,^^18^

A further application of sFC has been to investigate the neural coupling between two or more interacting individuals as a method for probing social interaction and aspects of the interpersonal relationship. Just as the relationship between fluctuations in activation within regions of the individual brain, both at rest and during tasks, may inform us of how these regions may integrate their functions, so too can the co-ordination of the neural time course between two individuals inform us of key aspects of their joint experience. The emerging studies that have examined synchronization of neural activation between two individuals have done so using hyperscanning^19^ (i.e., measuring the activation of interacting brains simultaneously). These studies have found preliminary evidence for interbrain coordination during regulated interactions^20^^–^^22^ and unrestrained social discourse.^23^^,^^24^

Although sFC methods were first demonstrated, and are still most widely employed, in functional MR BOLD imaging, similar networks have also been observed in other modalities including functional near-infrared spectroscopy (fNIRS).^25^^–^^28^ fNIRS is a noninvasive brain imaging technique that uses diffuse optical measurements in the red to near-infrared range (650 to 1000 nm) to measure hemoglobin changes resulting from fluctuations in cerebral blood flow and oxygenation.^29^ These signals are recorded between optical sources and detectors positioned on the scalp of a participant. Biological tissue is low absorbing but highly scattering in this range of wavelengths, which allows light from the source to penetrate through several centimeters of tissue along a diffuse path. This allows near-infrared light from sensors placed on the scalp to penetrate the outer layers of the head and skull and into the first few millimeters of the cortical surface. Changes to the optical properties along this diffuse volume between a source and detector position on the head alter the detected light signal and are reported as a time course of optical density (absorption) changes at each wavelength. Data at multiple wavelengths are then converted into estimates of oxy- and deoxyhemoglobin using the modified Beer–Lambert relationship.^30^ The reader is referred to several reviews on fNIRS theory,^31^ hardware,^32^^,^^33^ and applications^34^^–^^37^ for further details. Although fNIRS has lower spatial resolution than functional magnetic resonance imaging (fMRI) and only measures the surface of the brain, this technique is more portable, less costly, amenable to interactive paradigms, and more versatile to allow brain imaging in unique situations, including field testing, in-home, or clinical bedside studies due to its effective use in populations in which other neuroimaging methods are more challenging (e.g., infants, children, the elderly, or psychiatric patients). Thus, optimization of the sFC technique for fNIRS is critical for expanding the scientific and clinical knowledge of a wider breadth of normative and clinical populations.

The purpose of this paper is to describe the challenges to sFC-NIRS as they pertain to the statistical models, assumptions, and limitations of common analysis techniques for sFC. Specifically, there are several unresolved challenges to sFC-NIRS, including sensitivity to spurious correlations due to the slow hemodynamic signal, systemic physiological noise, and other measurement artifacts, such as subject motion. We describe and demonstrate how using an incorrect statistical model leads to high false-discovery rates (FDRs) in fNIRS analysis in the presence of such noise. FDR is defined as the number of false discoveries divided by the total number of discoveries (both true and false). In this paper, we first present an overview of the problem of spurious correlations and motion artifacts on fNIRS signals in the specific context of statistical models and demonstrate what effect these can have on the analysis and interpretation of sFC-NIRS. We then discuss the generalization of these statistical methods that make these models less sensitive to these sources of noise. After outlining the mathematical method used in this work, we provide quantitative comparisons of these approaches using both simulation results and experimental data. In this paper, we do not discuss preprocessing methods that may be used to reduce this noise. Rather, we focus on the problem from a statistical point of view in the context of generalized linear models (GLMs). We demonstrate that, even in the absence of spatially global systemic physiology, the autocorrelative structure of the hemodynamic signal results in substantial (>70%) FDRs at typical fNIRS sample rates.

## 2 Theory

Analysis of resting state connectivity in fNIRS is based on the statistical relationship of the spontaneous temporal fluctuations between two or more parts of the head (or two or more regions across subjects). Such analysis can be done in either the time domain (TD), for example, using correlation or causality models (e.g., Granger causality),^38^ or in the frequency domain (FD) using spectral coherence^39^ or phase-locking measurements.^40^ Spectral coherence is a measure of the relatedness of the amplitude and phase between two signals at specific frequencies or ranges. Phase-locking coherence is a measure of relatedness as a function of the phase (but not amplitude) information alone. In general, time-series models (correlation or Granger causality), which examine the relatedness of two slow hemodynamic signals over time, have historically been more popular in the sFC analysis of fMRI data.^41^^,^^42^ By contrast, the FD metrics of coherence and phase locking have been more widely used in electrophysiological recordings, such as electroencephalography and magnetoencephalography, where the frequency-specific values can provide insight into connections at specific neural oscillatory bands (e.g., the so-called alpha or beta rhythms).^7^ In fNIRS research, which has a higher acquisition rate than fMRI but still measures the slow hemodynamic signals, both TD (e.g., Refs. 43–47) and FD (e.g., Refs. 22, 48–50) approaches have been previously used.

### 2.1 Challenges to sFC-NIRS Connectivity

Both TD and FD analysis approaches to connectivity metrics typically make assumptions about the statistical properties of the underlying signals and its noise. Specifically, most standard analysis approaches, such as Pearson’s correlation, assume the noise in the signal to be independent, not self-correlated (zero autocorrelation), and normally distributed. As emphasized by Granger and Newbold,^38^ the presence of self-correlations in the signals can produce spurious correlations (false discoveries) across two channels of data. Specifically, spurious correlation is the concept that if two random signals that each have nonzero autocorrelation are compared, then the expectation of the absolute value of the correlation will be nonzero. Thus, it is essential to characterize the noise in the data when selecting and interpreting the results of a statistical analysis. In the context of fNIRS data, there are two main sources of noise that violate these assumptions of ideal noise structures—slow physiology and motion-related artifacts.

#### 2.1.1 fNIRS data exhibited autocorrelated, colored, noise structures

Since fNIRS signals are sensitive to slow physiological and systemic fluctuations due to cardiac, respiratory, and blood pressure changes, the noise in fNIRS is often highly structured. This physiological noise is structured and contains specific colored noise patterns around the frequencies of these systemic fluctuations (e.g., cardiac noise at 1 Hz, respiratory noise around 1/4  Hz, and slower blood pressure fluctuations). These slow physiological signals are present in both the superficial extracerebral layers and within the brain itself. In addition, even in the absence of global systemic physiological noise, the hemodynamic response in the brain is a slow physiological process, which takes time for the evolution of blood flow, volume, and oxygenation changes (e.g., the so-called “balloon model”).^51^ The impulse response function for a change in underlying “neural” signal is on the order of 8 to 12 s. Thus, even with the most sophisticated of preprocessing to remove nonbrain signals, the sources of noise and slow signals within the brain are typically still much slower than the sample rate of most fNIRS instruments. As a result, the noise in fNIRS data exhibits serially correlated errors, which means that the time points of the data have nonzero autocorrelation. For fNIRS, these vascular signals are typically oversampled resulting in an overestimation of the effective model degrees-of-freedom that often results in high FDRs.

The issue of serially correlated noise is a related but separate issue from the fact that much of this noise comes from global systemic physiology. In particular, these errors can result in statistical nonzero spurious correlations in the comparison of two unrelated signals with similar slow frequency content. As we demonstrate in this paper, there is a staggering 70% to 85% FDR (at a threshold of p<0.05 and 1-Hz sample rate) when calculating the correlations between two fNIRS time courses from different subjects on different days. This effect is due entirely to the slow nature of fNIRS noise and the resulting nonindependence of sequential sample points. Previous publications^52^^–^^54^ note the importance of accounting for these correlations in statistical modeling of evoked hemodynamic signals (e.g., functional brain data), and, in this publication, we demonstrate that these errors have a detrimental effect on sFC models as well. Fortunately, we also show that there is a fairly straightforward approach to correct this.

#### 2.1.2 Noise in fNIRS data exhibits temporal heteroscedasticity due to motion artifacts

In fNIRS, measurements are made between sources and detectors placed on the surface of the scalp. Although fNIRS measurements can be made during subject movement if the head cap is securely placed on the scalp (e.g., Ref. 55), motion-related artifacts will arise when one or both of these optodes move relative to the scalp. Often, this is the result of the probe sliding, releasing contact, or a change in the pressure applied to the probe in some way. In some populations, such as children and infants, where a looser fitting head cap is required for subject comfort and compliance, motion artifacts often occur. When the fNIRS probe transiently loses contact with the scalp, the signal may change by several folds beyond the normal level of noise in the data. Thus, motion will produce nonuniform noise distributions, which are often heavy tailed and contaminated by large infrequent jumps in the signal. These can be either spike-like artifacts, where the signal jumps but returns to the same value, or shift-like artifacts, where the signal returns to a new mean value. The variance of these data samples during and around an artifact can often be an order-of-magnitude above the variance of the regular noise in data. Thus, since the noise appears to arise from different distributions, this data exhibits heteroscedasticity.

**Fig. 1:**
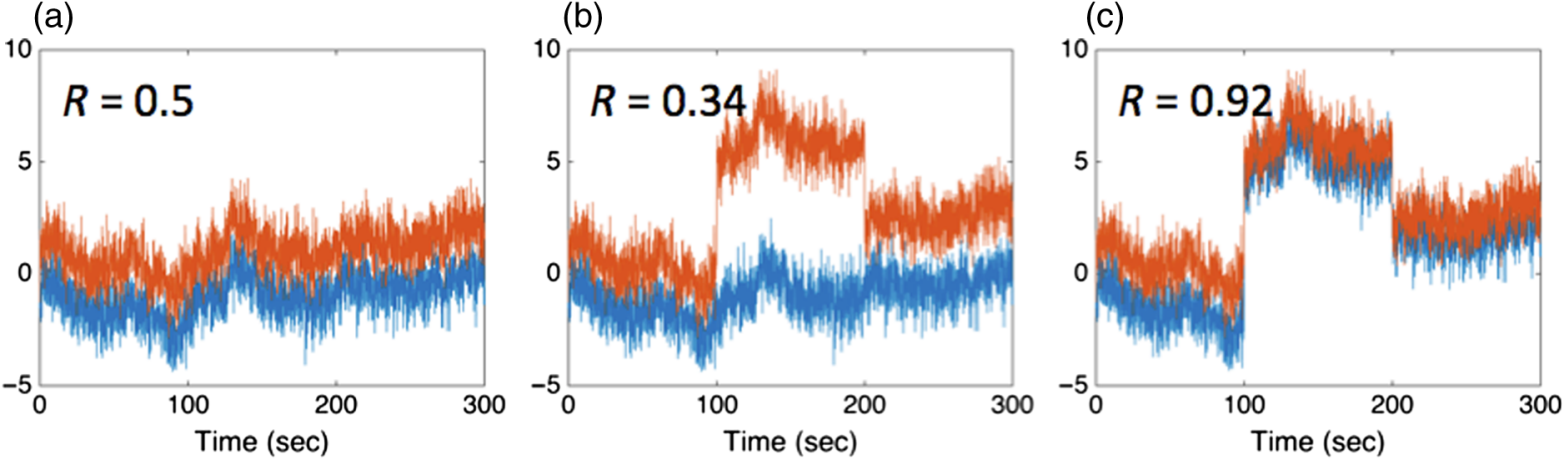
Demonstration of the effect of motion on fNIRS correlation analysis. In this figure, we show an example of simulated fNIRS data from two channels with a correlation of R=0.5 (panel a). If a motion artifact appears in one of the two channels (e.g., simulating the case where only part of the fNIRS probe moves), the motion dilutes the correlation and the connectivity is underestimated (panel b). However, if the motion artifact is co-occurring in both channels (e.g., the artifact has high spatial covariance), this can inflate the correlation and result in a high FDR (panel c) due to the strong leverage of the few affected outlier time points. (a) No motion, (b) unshared motion, and (c) shared motion.

Motion artifacts can lead to either high FDRs (type-I error) or high false-negative rates (type-II error) in correlation analyses depending on whether or not the motion artifact is co-occurring across multiple channels. Figure 1 shows a simulation of fNIRS data to demonstrate this property. In this demonstration, two fNIRS channels were simulated with a correlation of R=0.5 [Fig. 1(a)]. During fNIRS data collection with larger probes comprised of patches covering multiple regions of the brain, it is not uncommon to see motion artifacts that occur at different times for the different segments of the probe [Fig. 1(b)]. These artifacts arise from changes in the position or contact of the fNIRS probe. For example, facial muscles including the eyebrows can often create motion artifacts in fNIRS measurements over the forehead, which would not show up in measurements over the occipital cortex. Because the artifact causes a large jump in only some of the measurements, this dilutes the correlation due to the brain signals. In the simulated example to demonstrate this point, the correlation dropped from R=0.5 to 0.3 [Fig. 1(b)]. Other times, however, motion artifacts can co-occur across channels, as is often the case of physical movement of the subject, where the whole fNIRS probe shifts on the head [Fig. 1(c)]. In this case, the artifact drives up the correlation value; for this example, the correlation went from R=0.5 to 0.9 [Fig. 1(c)], which is an extremely significant but artificial increase (in this case, $p<2\times 10^{-300}$) in correlation. This shared artifact largely drives this high correlation, since motion artifacts are often about an order of magnitude larger in variance than the “brain” signals. Therefore, it is important to use statistical methods that discount outlier samples in the data. These outliers can either be univariate [present in only one of the two channels; Fig. 1(b)] or bivariate [present in both channels; Fig. 1(c)]. As described in this paper, the false significance can be correctly eliminated using a robust correlation method. The correlation for the data in this example is shown in Fig. 1 (R=0.50, 0.46, and 0.51), which, after the robust correlation method, are no longer statistically different (p=0.9). These methods are examined in a much more quantitative depth in this paper.

### 2.2 Generalized sFC Methods

To protect the sFC-fNIRS model from false-discoveries due to violations of the statistical assumptions in correlation, the noise model needs to be generalized to account for autocorrelative errors and motion-related outliers from a normal distribution. This is similar to the model generalizations needed for time-series regression analysis of evoked signal changes, which are implemented in the so-called GLM (e.g., Refs. 53 and 54). In particular, the term “general” in GLM refers to the generalization of the statistical model to relax the assumptions of independence, uncorrelated, and/or normally distributed properties of the noise. The GLM approach should be considered a collection of methods rather than a specific algorithm as it depends on specific statistical violations and properties that one is trying to generalize the model to handle. In this sense, the methods used for generalizing an fNIRS model require additional steps or higher-order corrections than those often used in other modalities, such as fMRI, because the properties of the noise in fNIRS are different. We have detailed and compared these approaches for evoked time-series analysis of fNIRS in a recent paper.^53^ In this section, we describe the similar preconditioning of fNIRS data for sFC connectivity. This section will briefly describe the mathematical concepts that we then apply in Sec. 4 of this paper.

#### 2.2.1 Prewhitening

Physiological noise results in temporally correlated (colored) noise in the fNIRS signals. This results in a reduction in the effective degrees-of-freedom of the data, since sequential sample points cannot be considered independent due to temporal autocorrelation. Prewhitening removes this autocorrelation and whitens the frequency content of the signal. The effect of this approach on fNIRS signals is presented in depth by Barker et al.^52^ In fMRI analysis, several papers have shown that prewhitening via autoregressive models is necessary to reduce false-discovery and greatly reduces the appearance of spurious global connectivity across the brain.^56^^,^^57^ A prewhitening filter can be defined from an autoregressive model of the data, which, once applied to the data, yields an uncorrelated innovations signal. The autoregressive model for a signal (Y) is defined as

$$Y_{\left\{t\right\}}=\overset{P}{\underset{i=1}{\sum }}a_{i}\cdot Y_{\left\{t-i\right\}}+\varepsilon_{\left\{t\right\}},$$ (1)

$$\varepsilon_{\left\{t\right\}}\in N(0,\sigma^{2}),$$ (2)

where {t} indicates the sample point (time) and the set $\left\{a_{i}\right\}$ is the autoregressive coefficients of the model, which need to be estimated. P is the model order, which can be selected using an information criterion, such as Bayesian information criteria (BIC).^58^ Equations (1) and (2) say that the current sample point ($Y_{\left\{t\right\}}$) can be predicted based on the last several time points in its history ($a_{1}Y_{\left\{t-1\right\}}\ldots a_{p}Y_{\left\{t-1\right\}}$) and newly added information at that time point, which is called the innovations ($\epsilon_{\left\{t\right\}}$). The innovations can be thought of as the new information that is added to the total signal at each time point. The innovations time course is a whitened signal with no autocorrelation representing the signal information added at each time point. The innovations signal can be estimated by first fitting the autoregressive coefficients of the model and using them to filter the original signal. Prewhitening is applied to both signals A and B to yield their respective whitened innovations models $A_{w}$ and $B_{w}$. Instead of correlating the original signals, the two innovations ($A_{w}$ and $B_{w}$) are compared, which estimate the correlation of the addition of only the new information being added to both signals at each time point. The so-called Granger causality models^38^ are an implementation of this concept that in particular look at the relationship of lagged cross-terms in the innovations (i.e., the history of signal B predicts the current value of A). In the remainder of this paper, however, we focus on the zeroth lag correlation terms.

**Fig. 2:**
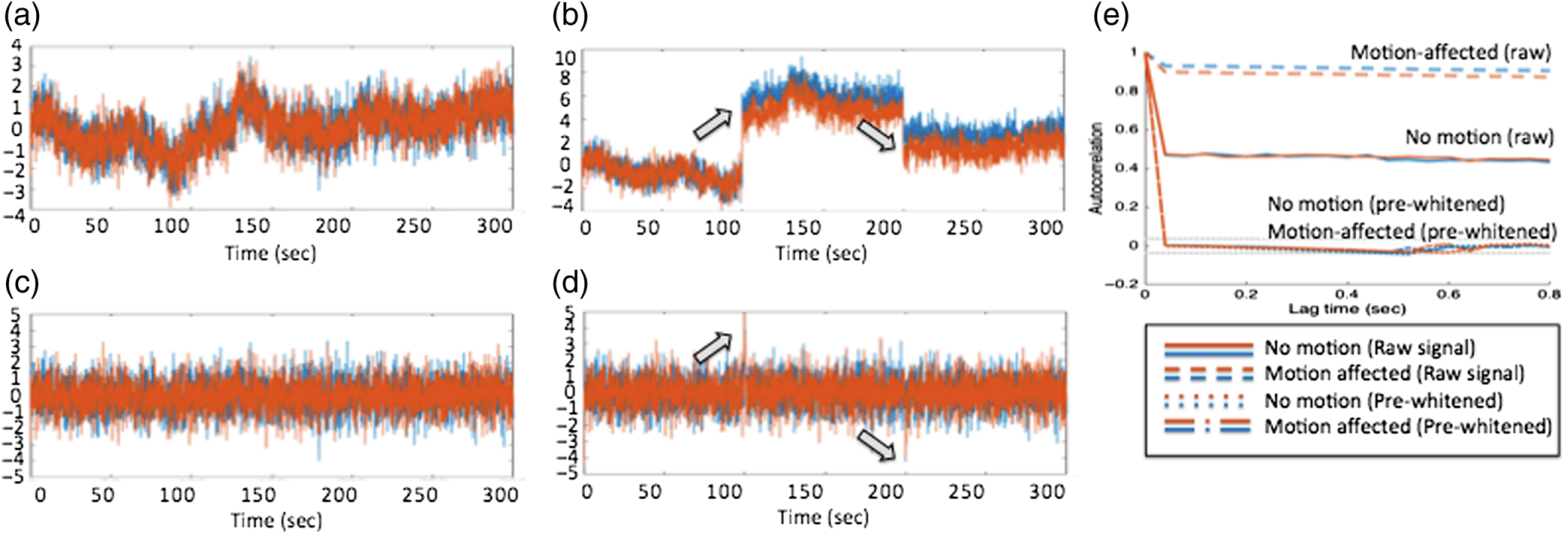
Effect of signal prewhitening on fNIRS data. Prewhitening using an autoregressive filter removes serially correlated errors in the signals. In panels (a) and (b), raw signals with no motion artifacts and with artifacts are shown. After filtering, the innovation (prewhitened) versions of these signals is shown in panels (c) and (d). This figure shows the same data presented in Fig. 1. (a) No motion (raw signal), (b) motion affected (raw signal), (c) no motion (prewhitened), (d) motion affected (prewhitened), and (e) autocorrelation.

Using the same simulated example from Fig. 1, Fig. 2 shows the effect of autoregressive whitening on the two signals, as shown in Figs. 2(c) and 2(d). In Fig. 2(e), the autocorrelation function is shown for both the motion-affected and -unaffected signals. The autocorrelation of the motion-affected data is considerably higher, but, in both cases, it is well above random chance for many tens of seconds of data. This means that the data points within 10 to 20 s of each other are not independent. After prewhitening using an appropriately high model order, the autocorrelation of both motion-affected and -unaffected drops to random chance within a single time point [Fig. 2(e)]. In real fNIRS data collected at around 5 Hz, based on BIC (depending on signal quality and sample rate), we found that autoregressive model orders of up to P=10 to 20 are generally needed to properly prewhiten the signals. We note that this is substantially higher than the models typically used in fMRI versions of this sort of analysis. In Sec. 4, we examine the effect of prewhitening in depth and compare the analysis of whitened and original data.

#### 2.2.2 Robust methods

Prewhitening via the autoregressive filter removes serial correlations and autocorrelation between sample points, but the whitened signals will still contain outliers (e.g., heavy-tailed noise) due to motion artifacts (see Ref. 53). As shown in Fig. 1, motion-related artifacts could be either co-occurring [Fig. 1(c)] or independent [Fig. 1(b)]. In our previous work in Barker et al.,^52^ we suggested that after prewhitening, both spike and shift type motion artifacts appear as isolated outlier points in the noise distribution. Figure 2 shows the same simulated signals shown in Fig. 1 after prewhitening. However, even after prewhitening, motion artifacts in fNIRS are often statistical outliers to the normal distribution compared to the rest of the unaffected data. The innovation signals during the motion artifacts (indicated by the arrows) deviate from the normal distribution. This can result in “high leverage” of these points in the mathematical model—a term that means that these outliers have a greater contribution to the estimate than other points.

The robust regression method (or preweighting) is an approach for dealing with statistical outliers in a linear model through iteratively estimating the residual noise of the model using a weighted least-squares fit and computing the weight based on outliers in the residual. Previously, this model was applied to reduce the effect of motion in fNIRS functional data.^52^ In this approach, the regression model is first solved and the residual noise from the data is estimated. Each time point of the residual is then studentized by normalizing to the standard deviation of the whole time course of the residual, so each time point can be assigned a probability of being an outlier. For example, the motion artifact shown in Fig. 2 is around 4 to 5 standard deviations from the rest of the noise and therefore associated with a low probability of belonging to the same distribution as the rest of the signal. A weighting function that downweights the residual to create a more normal distribution is then computed, this weight is applied to the original data, and the model is resolved. This is repeated until convergence. In MATLAB (Mathworks, Natick, Massachusetts), for example, this is implemented using the built-in function called “robustfit.” We have detailed the use of robust regression for investigating evoked signals in fNIRS in several of our previous publications.^52^^,^^53^

In robust regression, however, one signal is considered the data and the other is the regressor. Although correlation can be implemented as a regression problem, this assumes independent noise on the two channels. While this approach would fix leverage from motion artifact-related noises that act independently between channels [such as that in Fig. 1(b)], co-occurring artifacts would still result in false-discovery [as shown in Fig. 1(c)]. Therefore, we must introduce a bivariate version of this robust concept. We propose a joint weighting matrix, which is computed from the geometric length of both time courses, to reduce outliers and normalize the noise distributions, in Eq. (3):

$$r_{\left\{t\right\}}=\sqrt{A_{w\{t\}}^{2}+B_{w\{t\}}^{2}},$$ (3)

where $A_{w}$ and $B_{w}$ are the innovation (prewhitened) time courses of the data. This model can also be applied directly to the original data (A and B). Similarly, this equation can be extended in the case of multivariate models with three or more time courses. Time dependence is again indicated with subscript {t}. A weighting matrix (S) can be computed to downweight time points that are statistical outliers from the normal distribution. The same weight matrix is then applied to both of the data signals ($A_{S,W}=SA_{W}$ and $B_{S,W}=SB_{W}$). Thus, we propose computing the correlation between $A_{S,W}$ and $B_{S,W}$, which are now the two weighted and prewhitened signals, respectively. The weighting function (S) used in this work is given by

$$S\left(\frac{r}{\sigma }\right)=\{\begin{array}{cc} 1-{\left(\frac{r}{\sigma \cdot \kappa }\right)}^{2} & |\frac{r}{\sigma }|<\kappa \\ 0 & |\frac{r}{\sigma }|\geq \kappa \end{array},$$ (4)

which is simply the square root of Tukey’s bisquare function^59^ and is the same model as used in Eq. (4) from Barker et al.^52^ The tuning constant κ is typically set to 4.685, and σ is the standard deviation of the model error, which is estimated from the median absolute deviation (MAD) of the signal [σ=1.4826 MAD(r)]. The implementation of correlation estimates based on this approach is detailed in Sec. 2.3.

### 2.3 Computation of Functional Connectivity Metrics

Robust correlation approaches are used to control for the leverage of outliers. The review paper by Pasman and Shevlyakov^60^ describes a number of different approaches for robust estimates of correlation. In this work, we use a robust regression approach to estimating robust correlation,^60^ which closely follows the work we have previously proposed for analysis of functional time-series fNIRS data in Barker et al.^52^

The linear regression form of the correlation model for estimating functional connectivity between two measured signals ($A_{S,W}$ and $B_{S,W}$ following the notation from the previous section) is defined by the expression:

$$A_{S,W}=b_{0}+B_{S,W}\cdot b_{1}.$$ (5)

The two coefficients ($b_{0}$ and $b_{1}$) can be estimated by a least-squares solution to Eq. (5), and the correlation coefficient (R) is then given by

$$R=b_{1}\cdot \frac{\sigma_{A}}{\sigma_{B}},$$ (6)

where $\sigma_{A}$ and $\sigma_{B}$ are the standard deviations of the signals A and B, respectively. Equations (5) and (6) are also true for the converse of the model (regression of A onto B or B onto A should produce the same estimate of correlation for normally distributed signals). As an aside, this linear regression formulation of correlation for normally distributed errors is equivalent to the expectation form as given by

$$R=\frac{\mathrm{Cov}(A_{S,W},B_{S,W})}{\sqrt{\mathrm{VAR}(A_{S,W})*\mathrm{VAR}(B_{S,W})}}.$$ (7)

In this paper, we use the regression model since we feel that is easier to devise extensions needed to extend the mathematical notation to cover multivariate models, including Grangers causality. However, the review by Shevlyakov and Smirnov^60^ offers several other formulations to this robust correlation problem, which could be explored in future work.

The estimate of correlation by either Eqs. (5) or (7) assumes normally distributed and uncorrelated noise in both $A_{S,W}$ and $B_{S,W}$, and, as we have previously stated, these assumptions are often violated by systemic physiological noise and/or motion artifacts. The prewhitening step reduces the autocorrelation in the noise, and the preweighting step reduces co-occurring outliers from the distribution [e.g., the artifacts shown in Figs. 1(c) and 2(b)]. However, independent artifacts can still exist separately on each signal [Fig. 1(b)]; to compensate for this, we use a robust form of the regression model (weighted least squares). The matrix form of the regression–correlation equation [Eq. (5)] is given by

$$A_{S,W}=[\begin{array}{cc} 1 & B_{S,W} \end{array}]\cdot [\begin{array}{c} b_{0}\\ b_{1} \end{array}]\;\text{and}\;R_{B\to A}=b_{1}\cdot \frac{\sigma_{A}}{\sigma_{B}},$$ (8)

or it is equally valid as the converse

$$B_{S,W}=[\begin{array}{cc} 1 & A_{S,W} \end{array}]\cdot [\begin{array}{c} a_{0}\\ a_{1} \end{array}]\;\text{and}\;R_{A\to B}=a_{1}\cdot \frac{\sigma_{B}}{\sigma_{A}},$$ (9)

where for the robust model, the standard deviation (σ) was estimated from MAD of the signal. For ideal noise, both expressions produce equal estimates of the correlation coefficient, but, more generally, the correlation coefficient can be estimated by solving both expressions and using a combined estimate given by^60^

$$\|R\|=\sqrt{R_{A\to B}*R_{B\to A}},$$ (10)

where the sign of the correlation is estimated by either model (usually, they result in the same sign unless the correlation is not statistically different from zero).

The two separate models given in Eqs. (8) and (9) can be solved separately using an iterative robust regression algorithm (e.g., Ref. 52). This is done using an iterative approach in which the model Eq. (8) is first solved. For each model, the residual error (r) from the model fit is then computed and used to define a weighting matrix (S), which downweights time points that are statistical outliers from the normal distribution. This is identical to the step taken in preweighting but now is only based on one of the signals (A or B). The weight matrix is then applied to both sides of the model, yielding the expression:

$$S_{A}\cdot A_{S,W}=S_{A}\cdot [\begin{array}{cc} 1 & B_{S,W} \end{array}]\cdot [\begin{array}{c} b_{0}\\ b_{1} \end{array}],$$ (11)

with the equivalent form for the converse model [Eq. (9)]. The weighting function (S) used in this work is given by the same expression as in Eq. (4) with the standard deviation now defined from the residual of the A→B model (or the B→A model). Finally, the weighted model [Eq. (11)] is then resolved, new weights are computed, and the process is iterated until convergence. The two models are solved iteratively using this approach, and the separate estimates of the correlation coefficient are combined via Eq. (10).

## 3 Methods

In this paper, we compare the various methods for calculating functional connectivity described above and investigate the sensitivity and specificity of the methods as well as the FDRs of the models.

### 3.1 Numerical Simulations

We used three sets of simulations to look at FDR and sensitivity–specificity of these sFC-fNIRS methods. This section describes those three types of simulations.

#### 3.1.1 Investigation of false-discovery rates

In the first set of simulations, we looked at the FDRs of these various connectivity metrics in the presence of serially correlated error and/or motion artifacts. For these simulations, two random (i.i.d.) normally distributed signals were simulated using MATLAB (*randn* function). These two raw signals are denoted n(t) and are intended to represent two completely independent “neural” processes in the brain. By design of the random number generator, the two n(t) signals have zero expected correlation between them, and thus any correlation found represents a false-discovery. More specifically, at a threshold of p<0.05, we expect no more than 5% of the simulations to show correlation indicating proper control of type-I error. The random number generator used by MATLAB is indeed sufficient to satisfy this condition for these purposes. The two neural signals [n(t)] were then convolved with a canonical hemodynamic model to generate simulated hemodynamic signals [denoted h(t)]:

h(t)=n(t)*hrf. (12)

We used the hemodynamic response function (hrf), used commonly in the temporal linear regression model, for functional analysis (e.g., Ref. 54), although we note that the exact form of this hrf is not crucial to the interpretation of these results, and our qualitative conclusions are true for any low-pass filter (LPF) acting on the neural signals. After going through the hemodynamic response, the simulated data are temporally smooth and the noise within each channel is no longer uncorrelated. The sFC between these two simulated hemodynamic models [h(t)] is estimated by various algorithms detailed in the next section. These signals were simulated at different sample rates from 0.01 to 100 Hz while keeping the number of sample points (3000) constant between simulations. Simulations were repeated 2000 times for each condition.

To look at the effect of noise heteroscedasticity (motion artifacts) on the correlations, we ran a set of simulations, where additional normally distributed random noise with 10-fold higher variance was added to 5% of the samples of the neural signals [n(t)]. That is, 5% of the randomly selected sample points had additional noise added that was taken from a normal distribution with a variance of 10× the variance of the base simulation. This was based on the experimental data (described in Sec. 3.1.3) and reflects the simulations shown in Figs. 1 and 2. This created a heavy-tailed noise distribution in n(t). The same 5% subsample for both channels was used (case of co-occurring outlier “motion” noise), although the noise added to the two channels was independently distributed. That is, the higher level of noise was added to the same time points in both traces but was generated from independent samples of the distribution. Thus, the “motion” noise on the two channels had an equal chance of going in the opposite or the same directions. Similar to the other simulation, the neural signals were then convolved with the canonical hemodynamic model. The same numbers of simulations were performed as the nonmotion simulations.

#### 3.1.2 Investigation of sensitivity–specificity (simulation)

Sensitivity and specificity examine both the true-positive and false-negative discovery of the models. Receiver operator characteristic (ROC) curves were generated by simulating correlation in exactly half of the comparisons and computing the false-positive and true-positive rates for the various methods for comparison. In this second set of simulations, two random signals [n(t)] were generated from a multivariate normal distribution, where correlation is introduced from off-diagonals in the covariance model. Thus, half of the channel pairs had an expected nonzero correlation (positives). Like the previous simulation, the simulated “neural” signals [n(t)] were convolved with a canonical hemodynamic model to generate autocorrelated signals [h(t)] and resampled to 4 Hz. Sensitivity–specificity (ROC) curves were then calculated from the false-positive and true-positive rates of the models. We also estimated the control of the type-I error from the ROC curves by plotting the actual FDR extracted from the ROC curve against the reported p-value (expected FDR) reported by the algorithm (e.g., the number that “MATLAB reports” without correcting for the reduced degrees-of-freedom of the serially correlated data). Specifically, when there are violations to the statistical model, we expect the reported p-value to be less accurate and underestimate the false-positive rates leading to an uncontrolled type-I error.

#### 3.1.3 Investigation of sensitivity–specificity (experimental)

Finally, we examined sensitivity and specificity in experimental data to demonstrate that our simulations of motion and physiological noise in the previous simulations were experimentally appropriate. In the third set of simulations, experimental data from a separate study performed in children (ages 3 to 7 years old) were used. The collection of this data is described in Perlman et al.^61^ and contained 12 fNIRS source–detector channels of oxy- and deoxyhemoglobin from bilateral measurements of the forehead on 121 different subjects. This data contained both physiological noise and motion artifacts.

To examine the performance of the methods, a pair of channels was selected from either the same data file or different data files. Although the expected true correlation is unknown, we only expect nonzero correlations between data traces collected on the same subject at the same time. In other words, any correlation between two traces from different subjects (recorded on different days) would be a false-discovery. In each simulation, two data traces were randomly selected from the same subject’s data (nonzero correlation expected) or two different data files (null data). ROC analysis was performed to quantify the ability of the various models to differentiate between data that came from the same file and the data from different files (null distribution). Since, unlike the numerically simulated datasets, we were able to completely control the true-positive samples (e.g., some of the samples from the same file might not have actual experimental correlation and thus would be reported as a false-negative rather than a true-negative), this set of simulations allows us to look at the relative performances of the various methods but, due to the additional type-II error in the simulations, will underestimate the absolute sensitivity and specificity.

### 3.2 Comparison of Correlation Models

The three types of numerical simulations described in Sec. 3.1 were used to test the performance of several variations of the correlation models and preprocessing procedures. We then compared the results of several of the approaches outlined in Sec. 2.3. The analysis models that were used for comparison are listed below:

#### 3.2.1 Standard correlation model

In the standard model, we computed the Pearson correlation value by the standard equation. The correlation (COR) between two signals (A and B) was calculated without any preprocessing. This is the traditional estimate of correlation that does not include any corrections for serially correlated noise or motion artifacts (“corrcoef” function in MATLAB).

#### 3.2.2 Low-pass filtered correlation model

In previous work, analysis of the sFC network for fMRI or fNIRS has often been restricted to low frequencies (<0.1  Hz) by low-pass filtering the data prior to computing a correlation. In this analysis model, the data were first low-pass filtered using a backward/forward procedure (“filtfilt” function in MATLAB) and then the correlation was computed using the standard model.

#### 3.2.3 Preweighted correlation model

In the robust model, a preweighted regression model was used to estimate the robust correlation coefficient [preweighted correlation model (W-COR)]. However, no prewhitening was applied. This model uses Eqs. (8), (9), and (10) acting on the original data.

#### 3.2.4 Preweighted/prewhitened correlation model

In the AR-filtered model, serial correlations were removed with an AR filter for prewhitening and then the correlation was estimated using the standard model (“corrcoef” function in MATLAB). Finally, in the robust AR-filtered model, the full procedure (steps 1 to 7) was performed.

#### 3.2.5 Wavelet coherence model

Wavelet coherence (wCOH) was performed following the procedure described by Ref. 50 and using a modified version of the MATLAB toolbox developed by Grinsted et al.^39^ Cross-wCOH is given by the expression^39^

$$R_{n}^{2}(s)=\frac{{\left|W_{n}^{AB}(s)\right|}^{2}}{{\left|W_{n}^{A}(s)\right|}^{2}\cdot {\left|W_{n}^{B}(s)\right|}^{2}},$$ (13)

$$S[W_{n}^{X}(s)]=S_{\text{scale}}\{S_{\text{time}}[W_{n}^{X}(s)]\},$$ (14)

where $S_{\text{scale}}$ and $S_{\text{time}}$ denote smoothing operations along the wavelet scale (e.g., frequency) and time dimensions, respectively, of the wavelet transform and are given by the convolution of the wavelet matrix and smoothing operator (see Ref. 39).

#### 3.2.6 Prewhitened wavelet coherence model

In the prewhitened coherence model (AR-wCOH), the power spectrum of the data was whitened using an autoregressive filter prior to the continuous wavelet transform. wCOH was then computed between the two whitened signals, as given by Eqs. (13) and (14).

### 3.3 Implementation of Proposed Method

Our proposed robust correlation and FD methods were implemented in MATLAB 2015b as part of a toolbox for fNIRS research. This toolbox is currently available online and has been released open source at Ref. 62 or by request to the corresponding author. A demonstration script generating the figures shown in this work is included with the toolbox. wCOH models were based on modified versions of the models used in the MATLAB toolbox developed by Grinsted et al.^39^

## 4 Results

In this section, we present the results of the various versions of the analysis models applied to the three types of numerical simulations outlined in Sec. 3.1.

### 4.1 Investigation of False-Discovery Rates of fNIRS Correlation Methods

To begin looking at the problem of correlation in fNIRS, we first performed a simple simulation. Two normally distributed independent random variables were simulated at a sample rate of 0.01 to 50 Hz (3000 total sample points for each simulation), as detailed in Sec. 3.1.1. These two signals represent uncorrelated “neural activity,” which we denote n(t). The “hemodynamic” activity [h(t)] was then calculated by convolving n(t) with a linear canonical hemodynamic response [e.g., h(t)=hrf*n(t)]. The standard, robust, AR, and AR-robust models were then applied to compute the correlation between the two simulated “hemodynamic” signals [h(t)]. The AR-model order (up to ten times the sample rate in hertz) was determined by a BIC. In addition, we also looked at a bandlimited model in which the “hemodynamic” signals were low-pass filtered at 0.1 Hz using a fourth order Butterworth filter. This is a common procedure in fMRI and fNIRS to limit the calculation of correlation to only the low frequencies of the hemodynamic signal. As a negative control, we also computed the correlation for the original “neural” signals [n(t)]. This procedure was repeated 2000 times.

**Fig. 3:**
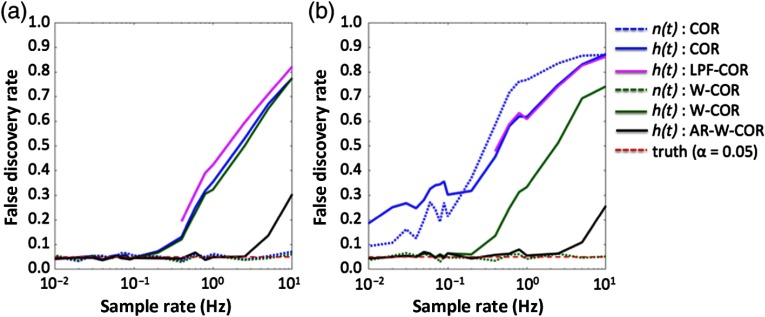
Comparison of FDR in simulated data as a result of sample rate. This figure shows the FDR for simulated “neural” [n(t)] and “hemodynamic” [h(t)] signals at different sampling rates and for the various correlation models described in Sec. 3.2. The expected FDR (α=0.05) is shown in the dotted red line. Estimates above this line are considered uncontrolled type-I errors. These simulations are described in Sec. 3.1.1. The abbreviations of the methods are defined in Sec. 3.2. (a) Without motion artifacts and (b) with motion artifacts.

Figure 3 shows the FDR of the various correlation models as a function of the sample rate of the simulated data. Since all simulations had the expectation of zero correlation (since they were generated from independent random variables), the FDR was calculated as the fraction of simulations, where the calculated probability was below p<.05.

Starting with the “neural” signals in the simulations without “motion” artifacts [Fig. 3(a)], both the robust and standard correlation models performed exactly as expected. At all sample rates, the FDR was around 5% at the p<0.05 threshold. By contrast, however, the simulated “hemodynamic” signals had a huge FDR introduced by the serial correlations from the canonical model acting as a LPF. At a sample rate of 1 Hz, the standard correlation model had a frightening FDR of 50% at the p<0.05 threshold. As the sample rate was decreased, the FDR went down but did not drop to 5% error until the sample rate was lower than the characteristic frequency of the canonical model (∼0.1  Hz). Low-pass filtering the “hemodynamic” signals prior to calculating correlation had very little impact on the model, but if a filter with a lower cutoff frequency was used (e.g., 0.05 Hz instead of 0.1 Hz), the FDR does not return to the 5% threshold until an even lower sample rate. The low-pass filtered signals are only shown at about a 0.2-Hz sample rate (since lower than this causes the passband to exceed the Nyquist frequency).

The proposed AR-filtered versions of the correlation model removed most of the serial correlations introduced by the canonical filter and produced FDRs close to the true value of 5% up to a sample rate of about 4 Hz for an AR-model order of up to P≤40 (determined by BIC). Above this sample rate, a higher model order was needed to remove the correlated temporal errors; however, this became computationally prohibitive above about 10 Hz for these simulations. For the simulations with no motion artifacts, the robust weighting had no effect on the results, although this also implies that it did not hurt the models to include this weighting even when it was not needed.

In Fig. 3(b), we show the results for the simulations with the addition of the 5% heteroscedastic noise to simulate motion artifacts in the “neural” signal. For the robust correlation methods, the results were similar to the simulations without motion artifacts. However, for the standard correlation model, the “motion” artifacts had a huge effect on the FDR. The standard model applied to the “neural” or raw “hemodynamic” signal had the largest errors with almost a 90% FDR. Thus, in the presence of motion artifacts, the robust procedures produced much more reliable control of type-I errors and insensitivity to the outlier noise due to the artifact.

### 4.2 Sensitivity and Specificity Analysis of the Models

**Fig. 4:**
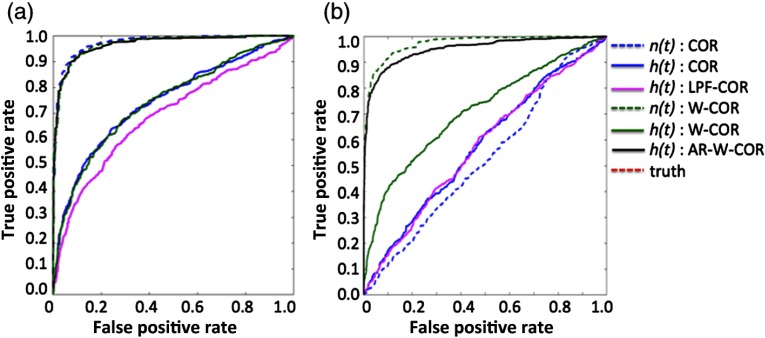
Comparison of sensitivity–specificity in simulated data. This figure shows the sensitivity–specificity (receiver operator curves) for the various correlation models applied to the simulated “neural” [n(t)] and “hemodynamic” [h(t)] signals. Curves that are closer to the upper left corner have better model performance. Panels (a) and (b) show simulations in the absence and presence of motion artifacts, respectively. These simulations are described in Sec. 3.1.2. The abbreviations of the methods are defined in Sec. 3.2. (a) Without motion artifacts and (b) with motion artifacts.

In this second set of simulations, we looked at the model performance of various approaches using numerically simulated data at 4 Hz. An equal number of numerically simulated signals was generated with and without correlation and was used to generate ROC curves to compare the various analysis models, as described in Sec. 3.1.2. Similar to the previous example, simulations were performed with or without the addition of 5% heteroscedastic noise (motion artifacts). The true-positive and false-positive rates were computed from these simulations to generate the ROC analysis (Fig. 4).

In the simulations without additional motion noise [Fig. 4(a)], the preweighting (robust correlation) and nonweighted (ordinary least squares) models had similar performance, which is consistent with the FDR results shown in Fig. 3. The prewhitened models have much better performance than the nonwhitened versions and were similar to the direct analysis of the neural signals [n(t)]. The nonwhitened procedure had about a 25% reduction in the area-under-the-curve of the ROC plots. Marginally, analysis of the 0.1 Hz low-pass filtered “hemodynamic” signal [h(t)] had slightly worse performance than the nonfiltered version and in both cases worse than the prewhitened or “neural” signals. Figure 4(b) presents the results from the signals simulated with additional 5% heteroscedastic noise to simulate motion artifacts. The addition of motion artifacts substantially decreased the sensitivity of both the “neural” and “hemodynamic” model but was recovered by the additional preweighting (robust correlation) procedures. Most affected was the analysis of the direct “neural” signal, which was reduced to near random chance using the nonweighted model but had similar performance to the simulations without motion if the robust correlation model was used. The prewhitened/preweighted model had similar performances with and without motion artifacts. The sensitivity of the model with motion artifacts was slightly decreased due to the additional noise added in these simulations. Preweighting (without additional prewhitening) did not improve the performance of the “hemodynamic” models and was actually worse than the standard correlation model. Low-pass filtering the “hemodynamic” response in the presence of motion artifacts also decreased the performance of the models.

### 4.3 Experimental Data (Time-Domain Models)

In this example, experimental fNIRS data were used, as described in Sec. 3.1.3. Two time traces were randomly selected from either the same data file (nonzero correlation expected; positive distribution) or different data files (no correlation expected; null distribution). The correlation between these two traces was computed using the standard correlation model, a 0.1-Hz LPF and then the standard correlation model, the robust model, and the AR-robust correlation model. The true-positive (correctly identified as coming from the same experimental data file) and false-positive (unexpected correction from two data in two different files) rates were determined for the four models.

**Fig. 5:**
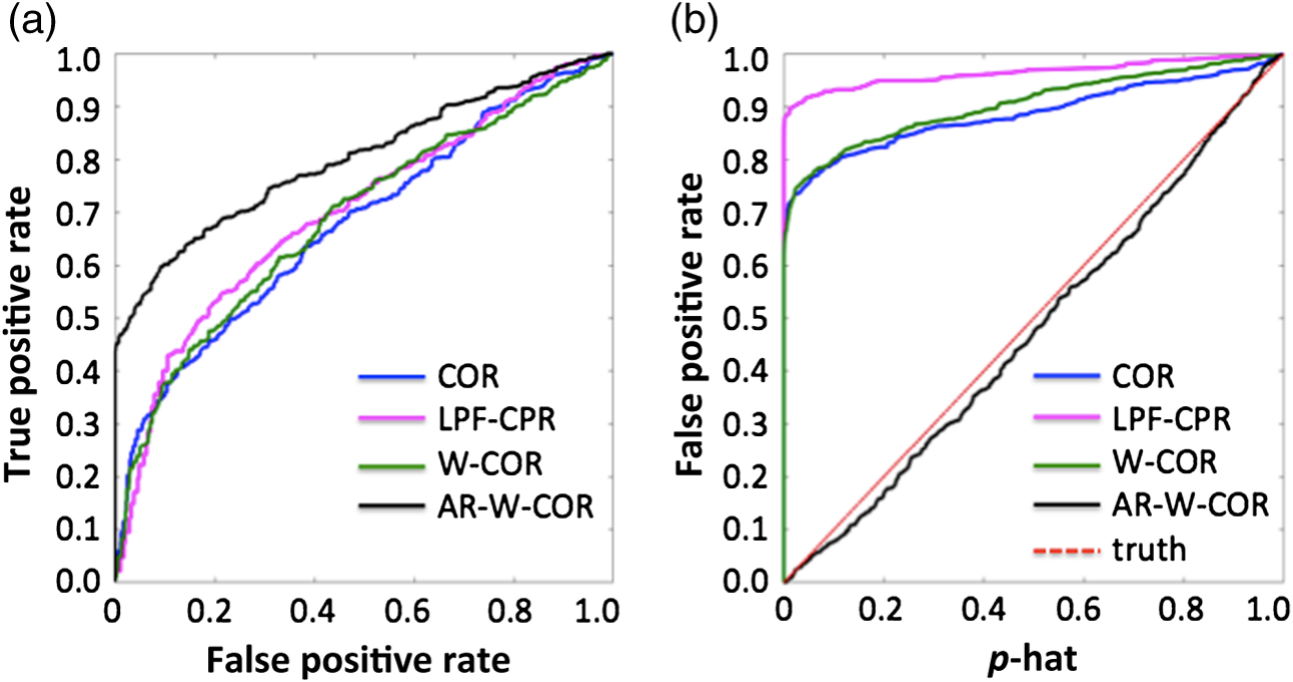
Comparison of sensitivity–specificity and type-I error control in experimental data. (a) The sensitivity–specificity (receiver operator curves) for the various correlation models applied to the experimental data described in Sec. 3.1.3. (b) Control for type-I errors for the same data and methods. The abbreviations of the methods are defined in Sec. 3.2.

Figure 5(a) shows the ROC curve for the four correlation models. In agreement with the simulation results, the robust AR model performed the best. In this example, we defined a true positive as correctly identifying that two channels came from the same file under the assumption that these two signals would have some nonzero correlation. Of course, not all channels from the same file will have strong correlation; this contributes to a less than perfect sensitivity (e.g., even under the best case, the true-positive rate will be less than 100%).

In this data, the true-positive rate controlled at a 5% false-discovery threshold was about 55% for the robust AR correlation model and only 20% for the standard and robust correlation models, meaning the AR model was more than twice as sensitive compared to the standard model.

**Fig. 6:**
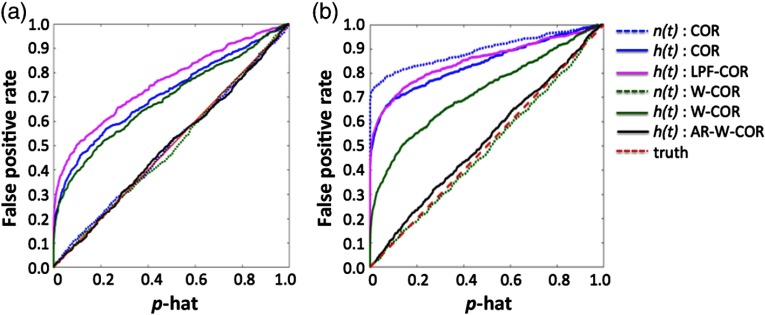
Comparison of control type-I errors in simulated data. This figure shows control for type-I errors for the various correlation models applied to the simulated “neural” [n(t)] and “hemodynamic” [h(t)] signals. The y-axis indicates the level of true false-discovery and the x-axis shows the reported probability (p-hat). An ideal curve would be along the diagonal (slope=1), where the reported and actual FDRs would be the same. Panels (a) and (b) show simulations in the absence and presence of motion artifacts, respectively. These simulations are described in Sec. 3.1.2. The abbreviations of the methods are defined in Sec. 3.2. (a) Without motion artifacts and (b) with motion artifacts.

Based on the ROC curve, we can also compute the true level of type-I error compared to the estimate provided by the model (p-hat; Fig. 6), namely, serially correlated error results in a decrease in the effective degrees-of-freedom of the data compared to the sample rate. This results in the reported p-value (p-hat) being artificially more significant than expected and uncontrolled type-I errors. In practical terms, this means that the value MATLAB reports (p-hat) using the standard correlation model can be substantially off from the actual false-positive rate because the serial correlations violate the assumption of independent uncorrelated noise in the data. In Fig. 6, the true false-positive rate is plotted against the reported value (p-hat). The robust-AR correlation model is very close to the ideal case (e.g., the reported value is correct). However, with uncontrolled serially correlated noise, the reported p-value of the model is extremely high. In particular, at a reported value of p-hat <0.05, the actual FDR is around 0.70 for the standard model and 0.85 for the 0.1 Hz bandlimited (low-pass filtered) correlation model. Without controlling for these serial correlations, this means that the p-value reported by the standard correlation method is completely inaccurate (wrong 70% to 85% of the time when displayed at a threshold of p<0.05). Note further that this is not a multiple comparisons issue since this inaccuracy applies to a single statistical test. For example, a Bonferroni correction adjusts for multiple comparisons; however, it assumes that the value for the single comparison test is valid, which is shown to not be the case for many of these correlation models. In the case of multiple comparisons (e.g., to form an all-to-all connectivity network), these type-I errors are even more greatly magnified.

**Fig. 7:**
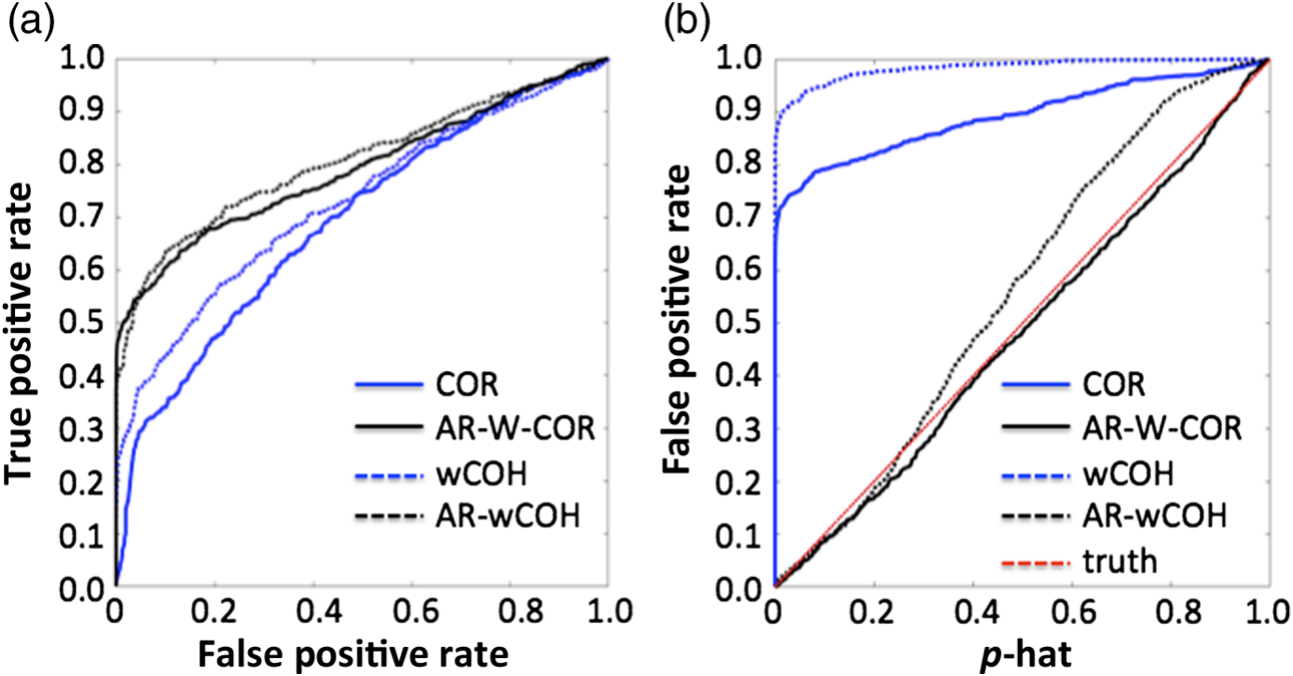
Comparison of sensitivity–specificity and control type-I error in experimental data. (a) Comparison of the performance of the unwhitened and prewhitened COR and wCOH models using the experimental data described in Sec. 3.1.3. (b) Control for type-I errors for the same data and methods. In panel (b), idealized control for type-I error is indicated by a line of unity slope (red dotted line). The abbreviations of the methods are defined in Sec. 3.2.

Figure 7 compares the sensitivity–specificity and control of type-I errors for FD coherence and TD correlation models from simulations based on the experimental data described in Sec. 3.1.3. For both the TD and FD models, autoregressive prewhitening had a substantial improvement on the control for type-I error rates. Similar to the previous finding, the (unwhitened) correlation model had about 75.2% false-discovery (at the 20 Hz sample rate with a p<0.05 threshold) while the wCOH model had an even higher false-discovery of 92.5% [Fig. 7(b)]. After autoregressive prewhitening, the false-discovery was controlled to 4.1% and 4.7% (expected 5%) although the coherence model deviated slightly from ideal at p-values greater than about 30% to 40% [Fig. 7(b)]. Likewise, autoregressive prewhitening improved the performance of both methods in analysis of the ROC curves [Fig. 7(a)]. The true-positive rate (at p<0.05) was 29.6% and 37.8% for the correlation and coherence models in the unwhitened case, respectively. This improved to 54.8% and 45.1%, respectively, with prewhitening. The area under the curve for the ROC plots was 59.6% (correlation) and 61.4% (coherence) for the unwhitened case and 64.5% (correlation) and 65.3% (coherence) for the prewhitened case. In both cases, prewhitening had a beneficial effect on performance, but both the correlation and coherence models appeared to have similar performances after whitening. Thus, we did not find any evidence to favor either of the correlation or coherence models over one another.

## 5 Discussion

In this work, we demonstrated [Fig. 5(b)] that, when we randomly selected two experimental time courses of fNIRS data from different subjects collected at different times, we found that 70% to 80% of the samples were falsely correlated at better than p<0.05. This is an indication of a clear problem with uncontrolled type-I error in the standard analysis methods, which have previously been used for sFC-fNIRS. Although this is a well-known problem that Granger described in 1974 as spurious correlations in signals with temporal self-correlation,^38^ this problem has been almost entirely neglected in fNIRS analysis. It is important to note that these high FDRs are not specifically the result of uncontrolled systemic physiological signals, but rather they occur because the hemodynamic signal of interest in the brain is inherently much slower than the typical sample rate of fNIRS instruments. For both evoked and spontaneous fluctuations in cerebral hemodynamic signals, the impulse response of the vascular system (the response to a negligibly short change in “neural” signals) is expected to be around 8 to 12 s long. This slow response is the result of the biomechanical expansion of blood vessels and oxygen transport dynamics (see Ref. 51). The presence of additional slow blood pressure, respiratory, and cardiac fluctuations further increases the level of serial correlations in the data; however, such a high false-discovery would be found even if these sources of additional noise were removed by preprocessing methods.

Our work in this paper is similar to previous reports for fMRI resting-state studies.^56^^,^^57^ In the paper by Christova et al.,^57^ they reported about a 20% FDR for fMRI at a 0.5-Hz sample rate, which is consistent with data presented in our Fig. 3. For fNIRS data, the typically higher sample rates of instruments make these false discoveries even more pronounced. For experimental data collected at 4 Hz, we found that FDRs as high as 70% at an expected level of p<0.05 indicated highly uncontrolled type-I errors in these estimates. These errors became worse if the signals were low-pass filtered as part of a preprocessing step and we found that the practice of low-pass filtering the data at 0.1 Hz produced uncontrolled errors as high as 90% in experimental data.

Importantly, this finding of high false-discovery is not specifically an artifact of the well-known global superficial contamination in fNIRS recordings. Rather, this is in addition to these global artifacts and should not be expected to be corrected by preprocessing methods. As evidence of this point, Christova et al.^57^ found the same result in fMRI. In addition, our simulations took experimental data from two different subjects collected on different days, so there is no expectation of a shared global systemic response. Thus, our conclusions that >70% FDR is serial-correlations that are not properly accounted for is expected to be a lower bound on FDR and the presence of uncorrected global and superficial signals would further increase this value.

Although this work focused specifically on continuous-wave (CW) fNIRS, these conclusions are based on the high FDR resulting from slow, autocorrelated errors general to the hemodynamic signal found both inside the brain (e.g., also seen in fMRI^57^) and from superficial contamination. Therefore, we feel that these results extend to both FD and TD variations of fNIRS as well. However, we note that with FD- and TD-fNIRS, the sampling rate of the instruments is generally lower; thus, serial correlations will have a lower but still significant effect on FDR for these modalities (refer to Fig. 3). This is also true of fMRI, which generally samples around 0.5 to 1 Hz. In addition, we note that as the relative contribution of random instrument noise to physiological signals increases, for example, due to poor coupling of sensors on the head or instrument design, the FDR from spurious correlations will decrease along with true-positive rates and sensitivity. Thus, FD- and TD-fNIRS may also have lower FDR rates then CW-fNIRS since the overall sensitivity of the methods and instruments is often lower. In addition, for TD-fNIRS, the use of “early photons” to provide information from the superficial skin layers can also provide a partial correction for systemic physiological signals. However, this does not correct the deeper noise signals from the brain. Similar corrections from multidistance source-to-detector pairs allow separation of skin and brain signals for CW- and FD-fNIRS methods. Even with these corrections, FDR rates similar or higher to those found in brain only regions with fMRI (20% for p<0.05 at 0.5 Hz)^57^ would be expected for all forms of fNIRS.

In our opinion, the methods proposed in this work should be considered as required steps in addition to any preprocessing methods to remove global nonbrain signals. Isolating the signals from the brain from these superficial fluctuations is a challenge and area of open investigation. Several methods have been proposed to address this issue, including the use of static-^63^^,^^64^ or dynamic-regression^63^^,^^65^ methods and independent^66^ or principal-component analysis.^66^ Some have suggested that short-separation measurements^67^ are the most direct approach to isolating skin and brain signals, and several recent publications have collected data using this method.^68^^,^^69^ This approach is based on the principal that the penetration of light into tissue is less for short-separation measurements; thus, measurements at distances of only 5 to 10 mm are used to capture the time-course of the skin, which is then regressed as covariate of no interest from the longer measurements that sense both brain and skin. However, there are several questions about how dense these measurements need to be and the optimal distance of these measurements, which has been examined by a few groups.^68^^,^^69^ The work by Brigadoi and Cooper^70^ used simulations from anatomical images to optimal separation distances and found that really short (∼2  mm) distances were recommended for infants, whereas longer distances (∼8  mm) could be used for adults. In practice, however, these really short distances (<1  cm) are often harder to obtain. Although really short measurements are sensitive entirely to skin, these measurements are more sensitive to motion of the fNIRS cap on the head because the light signal is typically brighter and more sensitive to the pressure of the fNIRS sensor. These are also affected by local heterogeneity in the vasculature of the skin (e.g., larger blood vessels), and the brightness of the light from short distances can often exceed the dynamic range of the photon detectors, particularly when trying to record both short and longer distances concurrently. Ideally, for these short-separation regression methods to properly clean the superficial noise, the short-distance measurement should be free of artifact and at least as high quality in terms of the signal-to-noise ratio as the longer distance measurement to be cleaned. When a short-separation regressor/data is noisy, this can actually transfer some of the noise into the signal of interest. Thus, using short-distance measurements that contain artifacts or high levels of noise as regressors can actually introduce additional error. Using short-separation measurements to remove physiology-based artifacts assumes that the same components are found in both the skin and brain. In a recently published paper,^71^ we found up to a 7-s delay between different measurements sites; this delay might be found between the skin and brain regions. Therefore, a simple regressor may not be efficient enough to remove the physiological noise. Secondly, we found that global changes in slow varying fluctuations accounted for only 25% of the spectral power in the low frequency band at the cortical level. In other words, main changes in low frequency fluctuations were related to the brain autoregulatory activities as well spatial variability due to vasculature differences. Therefore, short-separation measurements will not be that useful for removing physiology at the cortical levels.

## 6 Conclusion

Based on the simulations and analysis presented in this work, we conclude as follows:

• The slow evolution of the hemodynamic response results in spurious correlations, particularly at sample rates about 0.1 Hz, and results in uncontrolled type-I errors in the estimates of sFC.
• Autoregressive filtering to prewhiten the signals followed by correlation estimates of the innovations models provided control of these type-I errors and improved the sensitivities of the models in ROC curve analysis.
• We found that robust correlation of the innovations models provided more reliable estimates in the presence of motion-related artifacts.
• Prewhitening and robust methods improved the performance of both time series (correlation) and FD (wCOH) methods.
• We failed to find any strong evidence to suggest that TD or FD methods would be preferred and found similar performances of the two methods when compared in ROC analysis.

In conclusion, our results strongly suggest that accounting for both serially correlated errors and statistical outliers due to motion-related artifacts is essential to proper analysis and interpretation of resting state sFC-fNIRS signals.
